# Rapid evolutionary adaptation to elevated salt concentrations in pathogenic freshwater bacteria *Serratia marcescens*

**DOI:** 10.1002/ece3.1253

**Published:** 2014-09-23

**Authors:** Tarmo Ketola, Teppo Hiltunen

**Affiliations:** 1 Centre of Excellence in Biological Interactions, Department of Biological and Environmental Science, University of JyväskyläP.O. Box 35, Jyväskylä, FI-40014, Finland; 2 Department of Food and Environmental Sciences/Microbiology and Biotechnology, University of HelsinkiP.O. Box 65, Helsinki, 00014, Finland

**Keywords:** Experimental evolution, fluctuating environment, harsh environment, niche expansion, pathogen invasions, tolerance curve

## Abstract

Rapid evolutionary adaptions to new and previously detrimental environmental conditions can increase the risk of invasion by novel pathogens. We tested this hypothesis with a 133-day-long evolutionary experiment studying the evolution of the pathogenic *Serratia marcescens* bacterium at salinity niche boundary and in fluctuating conditions. We found that *S. marcescens* evolved at harsh (80 g/L) and extreme (100 g/L) salt conditions had clearly improved salt tolerance than those evolved in the other three treatments (ancestral conditions, nonsaline conditions, and fluctuating salt conditions). Evolutionary theories suggest that fastest evolutionary changes could be observed in intermediate selection pressures. Therefore, we originally hypothesized that extreme conditions, such as our 100 g/L salinity treatment, could lead to slower adaptation due to low population sizes. However, no evolutionary differences were observed between populations evolved in harsh and extreme conditions. This suggests that in the study presented here, low population sizes did not prevent evolution in the long run. On the whole, the adaptive potential observed here could be important for the transition of pathogenic *S. marcescens* bacteria from human-impacted freshwater environments, such as wastewater treatment plants, to marine habitats, where they are known to infect and kill corals (e.g., through white pox disease).

## Introduction

Human activity can expose microbial populations to novel and harsh environments (Logares et al. 2009). One such human-induced selection is leakage of potentially pathogenic bacteria to marine environments, usually with human origin and first leaked into the freshwater environment. During this process, freshwater pathogens will experience a potentially rapid increase in the surrounding salinity. Tolerance to withstand rapid increases in the salinity can be an important factor determining species distributions namely, salinity has been hypothesized to be a key factor in determining environmental distributions of microbes (Logares et al. 2009). This makes understanding adaptation of salt tolerance essential in order to understand the distribution of pathogenic bacteria in the freshwater/marine environment interface. Furthermore, one could hypothesize that an ability to overcome such strong selective barrier by evolution could promote the invasion of freshwater bacteria, some of which are pathogenic, to marine ecosystems. Despite the importance of salinity to the distribution of microbes, there have been only a few experimental studies on microbe adaption to saline conditions (Bell and Gonzalez 2009; Gonzales and Bell 2012).

Directional selection for greater tolerance of extreme conditions, such as high salinity, is expected to lead to improved performance in extreme conditions with faster and stronger selection. In this scenario, populations that have evolved in more extreme environments would have higher ability to tolerate these extremes compared with populations evolved in more subtle conditions (“sliding niche model”; Mongold et al. 1999). Naturally, it could also be that tolerance evolution beyond a certain point is not possible or that it is considerably slowed down, which would lead to no differences between populations regardless of the selection intensity. Such a case could occur when the available genetic variation is used up, and further adaptation needs *de novo* mutations. Related to this scenario, Mongold et al. (1999), see also (Bradshaw 1991) proposed also a “stepping stone model” of evolution which postulates that evolving first in the more benign conditions to acquire the needed mutations is a prerequisite for improved tolerance of more extreme conditions.

However, evolutionary changes depend not only on selection and heritable genetic variation in the selected trait, but also on the processes that create and maintain the selectable variation (Falconer and Mackay 1996). These latter phenomena are also very sensitive to population sizes, suggesting that a too strong selection pressure could lead to lack of evolutionary changes if it reduces population sizes below certain threshold levels (Robertson 1960; Gomulkiewicz and Holt 1995; Gonzales and Bell 2012, Ramsayer et al. 2013). Thus, if population sizes are relatively low, the likelihood of evolutionary change might be reduced, as the odds of obtaining the required mutations are also lower. However, in practice, it is empirically very hard to make distinction between (1) a weaker selective environment (in this case also the less extreme environment) promoting evolutionary effects due to higher population sizes and (2) environmental effects on the emergence of *de novo* mutations doing so (“stepping stone model”, see above).

Interestingly, fluctuating environments could offer partial resolution to the dilemma of strong selection leading to low population sizes by allowing bouts of strong selection to extreme conditions and recovery of population sizes at optimal conditions (Bell 2012). We are not aware of theoretical models explicitly exploring the feasibility of this idea. Nevertheless, in principle, fluctuating environments could promote dominance by genotypes adapted to extreme environments when novel genetic variation is present and it is useful for populations when facing extreme conditions. In extreme conditions, selection then lowers the frequency of nonadapted genotypes, and when returned to optimal conditions, “extreme genotypes” would not pay too high a cost. This scenario would give “extreme genotypes” an overall fitness benefit over the range of environmental conditions, increasing their frequency over time and simultaneously increasing the populations' overall tolerance to extreme environments. There is already some experimental evidence that any costs of being adapted to extreme environments are not suffered in optimal conditions, (Collins and Bell 2004; Hall and Colegrave 2008; Ketola et al. 2013). However, evidence for this proposed scenario would require extensive modeling. As environmental conditions in nature are rarely stable but fluctuate often between optimum and adverse, testing the role of variable environmental condition on niche evolution is important. Several types of fluctuating environments have been investigated (see Kassen 2002 for review), such as temperature (Leroi et al. 1994; Ketola et al. 2004, 2013), light (Kassen and Bell 1998); pH (Hughes et al. 2007), and resources (Buckling et al. 2007; Jasmin and Kassen 2007). To our knowledge, however, adaption to fluctuating saline conditions has not yet been explored.

To explore roles of extreme and fluctuating conditions on niche evolution, we conducted a replicated (*n* = 3 in stable conditions and *n* = 9 in fluctuating conditions) evolution experiment where a facultative pathogenic strain of *Serratia marcescens* freshwater bacteria evolved in four treatments: nonsaline (0 g/L), harsh (80 g/L), extreme (100 g/L), and variable (weekly changing) NaCl concentrations. After 133 days, we extracted altogether 90 individual clones (genotypes) from experimental populations and measured their growth performances in different salt conditions. During the experiment, the biomass of bacteria was measured during the weekly renewal of resources. This setup allowed us to explore the bacteria's adaptation potential to high salt concentrations and whether strains with stronger selection tolerate greater high salt concentrations. In addition, we could test whether the fluctuating environment selected for improved ability to tolerate several salt concentrations than constant salinity environments, as is often expected (Kassen 2002). Or if alternatively, whether fluctuations led to a better evolutionary response to selection and thus high ability to tolerate very high salt conditions. We found strong evolutionary adaptation even to extremely saline conditions. This suggest that evolutionary capability to tolerate high salinities combined with an increased human-induced leakage of *Serratia marcescens* and other freshwater pathogenic bacteria to marine environment could allow these species to become problematic to the marine ecosystem. We hypothesize that a rapid evolution in salt tolerance has the potential to cause otherwise sporadic occurrence of epidemics caused by freshwater pathogens in saline environments to become more frequent and severe.

## Materials and Methods

### Study organism

As a study organism we used heterotrophic bacteria, *Serratia marcescens* (from American Type Culture Collection strain ATCC 13880), that is a gram-negative, rod shaped bacteria that do not form spores. *S. marcescens* is facultatively anaerobic, typically 0.3–1.0  ×  1.0–6.0 *μ*m bacterium, and belongs to the family of Enterobacteriaceae (Grimont and Grimont 1978, 2006; Krieg and Holt 1984). In optimal condition, generation time can be less than 1 h (Fedrigo et al. 2011). The ATCC 13880 strain of *S. marcescens* was originally isolated from pond water, and species can be found in soil and in aquatic environments (Sutherland et al. 2010; Mahlen 2011). In addition to free-living life style, *S. marcescens* is also an opportunistic pathogen infecting a broad spectrum of hosts, including plants, corals, nematodes, insects, fish, and mammals (Grimont and Grimont 1978; Flyg et al. 1980). This strain of *S. marcescens* has been used also as a model organism in previous studies exploring the evolution of virulence (Friman et al. 2009, 2011).

### Selection experiment

In order to select populations with different evolutionary histories regarding the salt tolerance, we conducted a 133-day-long microcosm experiment where bacterial populations were exposed to extreme and also fluctuating environmental conditions. All treatments were started with a single ancestor colony of *S. marcescens* (in order to minimize the initial genetic variability in the population). This clone was also frozen and used as control for the selection treatments. In the long-term experiment, we manipulated salinity (harshness) of the environment and the temporal fluctuations in the salinity. Populations were cultured in three stable salinities: 0 (control), 80, and 100 g of NaCl in 1 liter of dH_2_O, and all treatments were replicated three times. To generate a fluctuating selection environment, we cultured nine replicate populations in an environment where salinity fluctuated between 0, 20, 40, 60, 80, and 100 g of NaCl in 1 liter of dH_2_O between each weekly transfer. Temporal changes in the salinity was a random process, and the time series were generated with an R script. Mean salinity in fluctuating environment was 49.5 ± 5.5 g/L.

As microcosms, we used 25 mL glass vials containing 5 mL of PPY culture media (PPY; 20 g of proteose peptone and 2.5 g of yeast extract in liter of dH_2_O). NaCl was added to PPY according to the selection environment treatment, all chemicals from Sigma-Aldrich. Every 7 days, vials were carefully mixed with vortex and 2% (100 *μ*L) of the culture was transferred into a new vial containing fresh culture media. All microcosms were kept in 28°C (±0.1 °C) with 50 rpm constant shaking. During each transfer, *Serratia* biomass was estimated as an optical density at 600 nm wavelength with UV-1800 spectrophotometer (Shimadzu, Japan) and a 0.5-mL subsample was frozen with 0.5 mL glycerol and kept in −80°C freezer for later analysis.

### Measuring evolutionary changes in the salt tolerance

After the long-term selection experiment, corresponding roughly 300 generations on average, we isolated five randomly selected clones from the each population. Thawed samples were first diluted and cultured on nutrient agar (20 g proteose peptone, 2.5 g yeast extract, and 15 g agar in liter of dH_2_O) for 48 h. Then, individual colonies (clones) were picked from the agar plates and stored in 50% glycerol in -80°C on a 100-well plate where each well contained a single clone placed in a randomized location in the well plate in order to avoid effects that well location might have on bacterial growth. Fitness measurements were initiated by replicating ca. 10 μl samples from frozen plates with cryo-replicator (Duetz et al. 2000) in to a new 100 well plate filled with fresh, non-saline culture media (400 μl. per well). This plate was then inoculated for 24 h in 28°C (±0.1°C). It must be noted that incubation for 24 h in these conditions corresponds to around twenty bacterial generations in nonsaline environment, indicating that our fitness results are in fact resulting from an evolutionary change rather than some induced mechanism or potentially different physiological states of the test populations. Moreover, this allows measurements without effects of glycerol residues from frozen stocks. After the initial 24-h incubation, a 10-*μ*L sample was transferred to a new plate containing fresh culture media with the desired test salinity. Growth of each clonal population was then monitored with Bioscreen C spectrophotometer (Growth Curves AB Ltd, Helsinki, Finland) where optical density of each well was measured at 480–580 nm wave length until all populations had reached the carrying capacity. As a metric for salt tolerance, we used maximum biomass, measured as optical density, which each population had reached.

### Data analysis

Evolutionary differences between the clones from different evolutionary treatments were analyzed with linear mixed models utilizing REML In analysis evolutionary treatment, measurement salinity and their interaction were fitted as fixed factors. To control for the nonindependency of the observations due to the clones originating from same source population, the identity of the population was fitted as a random factor. Repeated measures ANOVA (RMANOVA) was to analyze the population data during the selection experiment. Post hoc Tukey comparisons were performed to test pairwise interactions. All analyses were conducted with SPSS (SPSS Inc., Chicago, IL v. 20.0).

## Results

### Ecological dynamics during the selection experiment

**Figure 1:**
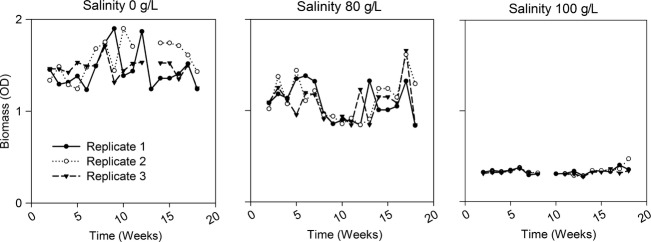
Weekly population biomasses (optical density) during the selection experiment in stable environment treatments. Note that few missing data points are due to lost samples during the sampling process.

**Figure 2:**
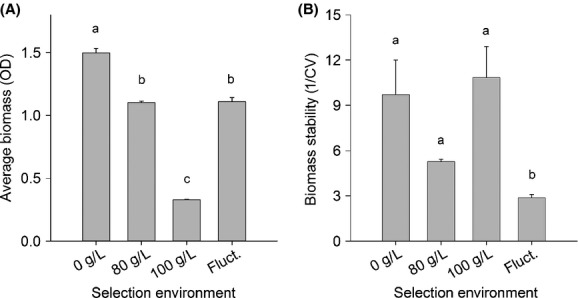
(A) Average biomass (optical density) ± SE in different salinity treatments during the selection experiment and (B) stability of the population biomass. Letters a–c indicate homogenous subsets that are not different from each other.

During the selection experiment, the average biomass in the 0 g/L salinity environment was higher than in all others, 80 g/L and fluctuating environment did not differ from each other, and average biomass in 100 g/L selection treatment was lower than in all other treatments (Figs.1, 2A and supplement Fig. S1 for biomasses in the fluctuating environment, *F*_3,14_ = 120, *P* > 0.001, pairwise comparisons: 0 vs. *F P* < 0.001; 0 vs. 80 *P* < 0.001; 0 vs. 100 *P* < 0.001; *F* vs. 100 *P* < 0.001; 80 vs. 100 *P* < 0.001). Stability of the population biomass was lower in fluctuating selection environment than in any of the stable environments (Fig.2B, *F*_3,14_ = 120, *P* > 0.001).

### Evolutionary change in the salt tolerance

We found that evolutionary changes occurred both on the elevation of the salt tolerance curve (Evolutionary history: *F*_3,278.083_ = 5.48, *P* = 0.001) and in the shape of the tolerance (Evolutionary history by Measurement environment interaction: *F*_12, 416.810_ = 5.72, *P* < 0.001). Measurement environments caused the strongest effects on the yield (*F*_4, 416.816_ = 4857, *P* < 0.001). Population identity, nested within the evolutionary treatment, indicated that evolution proceeded similarly within evolutionary treatments (Wald *Z*: 1.28, *P* = 0.202).

When ancestor clones were included in the dataset, we found that differences between average performances across all measurement environments (elevation of tolerance curve) were more pronounced (Evolutionary history: *F*_4, 323.823_ = 10.14, *P* < 0.001), and correspondingly the shape differences lessened (Evolutionary history by Measurement environment interaction: *F*_16,434.810_ = 4.75, *P* < 0.001). Again, measurement environment dictated differences in yield (*F*_4,434.803_ = 3884, *P* < 0.001). Population differences within evolutionary histories were statistically nonsignificant (Wald *Z*: 1.28, *P* = 0.200).

Averaged across all of the measurement environments, the clones evolved at nonsaline conditions had lowest yield (0.55), which was significantly lower than yield in clones evolved at 80 g/L (0.71, *P* = 0.02), in clones evolved at 100 g/L (0.79, *P* = 0.03), and in ancestral clones (0.70, *P* < 0.001). Clones from fluctuating environment did not deviate in their average performances (0.60) across the measurement environment from clones that had evolved at nonsaline conditions (*P* > 0.9). In pairwise testing, the clones from fluctuating environments had also lower performance than ancestor strains (*P* = 0.012). All other pairwise comparisons were clearly nonsignificant (*P* < 0.2).

Regardless if the data analysis was performed with full dataset or without ancestor clones, the result is the same. Due to Bonferroni corrections in pairwise comparisons, the p values are stronger if smaller dataset (without ancestor) is used. However, those pairwise comparisons that were found significant with smaller dataset were the same that were found significant in full dataset.

**Figure 3:**
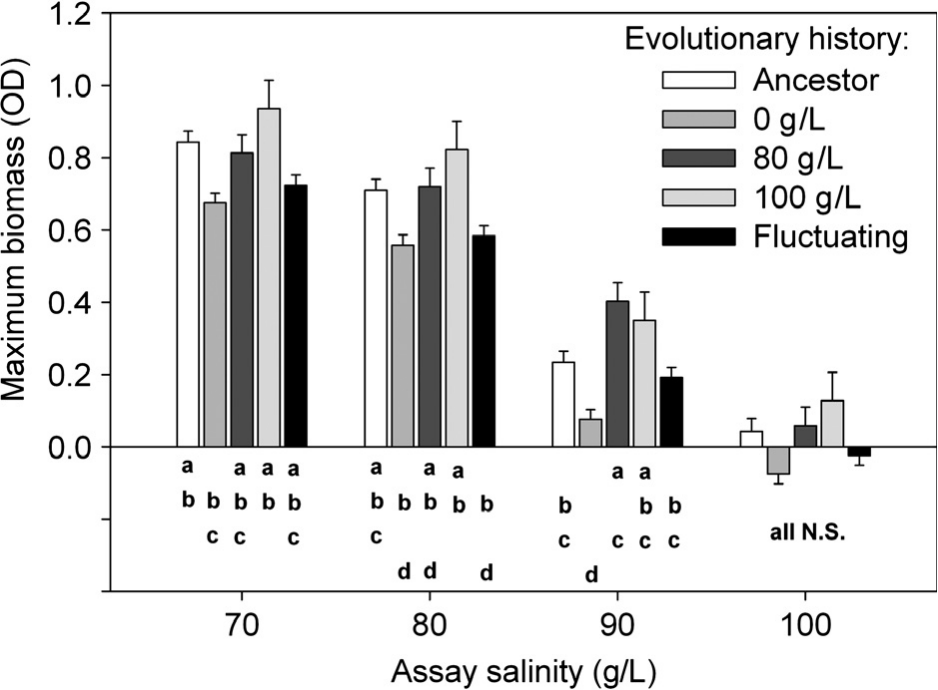
Evolutionary changes in tolerance to different levels of salinity measured by maximum obtained biomass (optical density) ± SE. Letters **a–d** indicate homogenous subsets based on pairwise comparisons of yield of differentially evolved clones (ancestral, 0, 80, 100 g/L and fluctuating salt concentrations) in different measurement salt concentrations. Note that all pairwise comparisons are corrected for multiple comparisons with Bonferroni correction.

Evolutionary history by measurement environment interaction was examined in more detail, and we found that the evolutionary differences were lowest in nonsalty conditions (full data: *F*_4,357.797_ = 3.86, *P* = 0.004, no ancestors: *F*_3,291.66_ = 2.00, *P* = 0.114) and in extremely salty conditions (full data: *F*_4,358.764_ = 3.30, *P* = 0.011; no ancestors: *F*_3,291.779_ = 3.17, *P* = 0.025). In both datasets, we found significant differences between clones' evolutionary histories in measurement salt concentrations of 70 g/L (full data: *F*_4,357.812_ = 6.22, *P* < 0.001, no ancestors: *F*_3,291.229_ = 4.48, *P* = 0.004) and 80 g/L (full data: *F*_4,356.669_ = 6.17, *P* < 0.001, no ancestors: *F*_3,292.074_ = 5.18, *P* = 0.002). By far, the largest differences between evolutionary histories of the clones were found at salt concentrations of 90 g/L (full data: *F*_4,357.758_ = 10.5 *P* < 0.001, no ancestors: *F*_3,291.181_ = 12.6, *P* < 0.001). We did not find any differences between evolutionary treatments in growth when assay medium did not contain salt. However, if ancestor was in comparisons, we found that ancestors' biomass was higher (mean biomass 1.64) than clones from nonsaline conditions (mean: 1.50, *P* = 0.017) and clones from fluctuating conditions (mean: 1.51, *P* = 0.032), and ancestors did not differ from biomass of clones evolved at 80 g/L (mean: 1.57) or at 100 g/L (mean: 1.68). Other pairwise comparisons are depicted in Fig.3 and in Table S1. When tested in nonsaline environment, no differences were observed between selection lines (mean maximum biomass ± S.E; ancestor; 1.65 ± 0.010, evolved strains: 0 g/L; 1.63 ± 0.003, 80 g/L; 1.61 ± 0.002, 100 g/L; 1.61 ± 0.012, variable environment; 1.63 ± 0.015, *F*_4.14_ = 0.91; *P* = 0.48).

## Discussion

Generally, evolutionary changes are expected to be stronger when selection is stronger, as it is predicted by the breeders equation (Falconer and Mackay 1996). However, in the case of adaptation to extreme environments, strong selection pressure can be associated with reduced population sizes. The latter can lead to lowered supply of adaptive mutations which could ultimately hinder evolutionary adaptations (Robertson 1960; Gomulkiewicz and Holt 1995; Falconer and Mackay 1996). This line of thought suggests that the conditions for the evolutionary adaptation could be more favorable under intermediate selection pressures that allow high population sizes and thus larger evolutionary potential. However, even though we succeeded manipulating the population size of our experimental populations closest to the salinity–niche border, we did not observe any difference in salt tolerance between clones evolved in harsh (80 g/L NaCl) versus extreme conditions (100 g/L NaCl) (Fig.3). However, these two groups of clones outperformed those obtained from fluctuating salt conditions and those from nonsaline conditions.

Theories suggest that evolutionary adaptation to harsh environmental conditions is most likely to occur in intermediate selection pressures (Robertson 1960; Gomulkiewicz and Holt 1995): where selection is sufficient but still permitting high enough population sizes and sufficient amount of new beneficial mutations. In contrast, we did not observe any evolutionary difference between the clones that had evolved in the two highest salt concentrations (80 and 100 g/L). It is noteworthy that even though population sizes were larger in the group with 80 g/L salt concentration compared with 100 g/L concentration, the long duration of our experiment would have allowed still enough mutational input for evolutionary change. Likewise, relatively long duration of the experiment could also explain why our fluctuating environment treatment did not seem to alleviate the problems of high selection combined with low population sizes (see also below). One scenario is that the same population can experience high selection pressure and large population when environmental conditions fluctuate. For example, when environmental conditions have been favorable, the population size is large, so that when the environment conditions change to less than optimal, the population may have larger standing stock of genetic variability in order to overcome this change. On the basis of this line of thought, one of our hypotheses was that fluctuating environments could lead to elevated salt tolerance. One potential explanation for why we did not see differences between harsh and extreme conditions and why clones from the fluctuating environment treatment performed relatively poorly could be that despite a significantly smaller population size in the 100 g/L treatment (cf. 80 g/L), it was still large enough. In their recent meta-analysis of published microbial experiments, Hiltunen et al. (2014) found that only c. 10^5^ individuals are needed for rapid evolutionary change to occur. Our estimate of total population sizes was several orders of magnitude larger, even in the 100 g/L treatment, thus well above the critical population size limit and the reason why the two highest salinities did not differ. Furthermore, the biomass in the fluctuating environment and in the 80 g/L selection treatment did not differ from each other. However, the evolutionary response to high salinities was very different in these two treatments. From the latter, we can conclude that in our experiment, selection strength was a more important factor in defining the evolutionary outcome than was population size. In previous evolutionary rescue experiments, population size has been found to cause a strong effect on the possibility of new mutations for survival in new conditions. However, it must be noted that in these studies, the selection strength was very high, and survival monitored over a very short period of time (Bell and Gonzalez 2009; Ramsayer et al. 2013). This leads us to believe that low population sizes simply slow down the emergence of evolutionary novelties, which might not be visible at the end of longer-term studies. Nevertheless, population size could be a very important factor in the short-term and during extremely strong selection bouts.

When we compared across treatments, we did not find differences in the degree of specialization to salt conditions between the two high salt concentration treatments. It appears that clones from high salt concentrations always do better regardless of the salt conditions where their growth was assayed. This suggests that specialization between two high salt concentrations might be prevented if we assume the “sliding niche model of evolution” (Mongold et al. 1999) where average salt tolerance dictates the level of adaptation. Thus, an increased salt tolerance at harsh concentrations will also increase the bacteria's tolerance to more extreme conditions and vice versa. As the clones from fluctuating environments did worse than the clones from high salt concentrations, selection on the mean of the fluctuations (∼50 g/L) could have been a stronger denominator in their adaptation to fluctuating salt conditions than any experienced extreme conditions. Unfortunately, we did not include a constant salt condition of 50 g/L treatment to test the latter. Nevertheless, our observations suggest that fluctuating growth conditions did not select for generalism, at least at the salinity levels we tested. This finding is in contrast to several studies where evidence for an evolved generalism was found to be the consequence of fluctuating environments (Leroi et al. 1994; Scheiner and Yampolsky 1998; Kassen and Bell 1998; Kassen 2002; Buckling et al. 2006, Ketola et al. 2013, 2014; Condon et al. 2014) One explanation for the lack of expected adaptation in fluctuating environments in the current study could be that our salinity fluctuations were too coarse-grained: a salinity change once a week might have turned our fluctuating environment into a series of stable environments. In fact, such environments are supposed to mostly select for specialists, whereas fine-grained environmental variation should give fitness advantage to generalist genotypes (e.g., Levins 1968). It is also possible that clones from fluctuating environment might have adapted to the most profitable environment or to the most commonly experienced environment (Jasmin and Kassen 2007). Moreover, clone performance in nonsaline conditions was equivalent across all treatments. Overall, the strains from fluctuating environments did considerably worse than any others tested, which clearly deviates from the common knowledge stating that fluctuating environments produce generalists. However, it must be noted that the main emphasis of this study was to explore evolution at extreme conditions and not to compare across a whole range of salt conditions.

By manipulating the salt concentration and salinity fluctuations, we show that *S. marcescens* can adapt to high salt concentrations. The two harshest conditions did not differ in their levels of salt tolerance. And despite the c. 3.5-fold biomass difference, this suggests in both treatments population sizes were large enough not to constrain bacterial evolution in the long run. Furthermore, populations kept in a fluctuating environment had equivalent population densities as populations from the 80 g/L treatment and regularly experienced extreme salinities. However, they still did not evolve to withstand extreme salinities. All in all, our results indicate that selection pressure is a more important factor driving salt tolerance adaptation in bacteria than is population size.

Our results have also more applied value due to the pathogenic nature of our study species and its known ability to inhabit the freshwater/marine environment boundary. Our evolutionary experiment clearly showed the evolution of higher salt tolerance in populations nearer to the upper end of their salinity niche that is in harsh or extreme salt conditions. This finding parallels the observation of fast evolution of bacteria when exposed to therapeutic concentrations of antibiotics, sometimes leading to the emergence of antibiotic-resistant pathogens (Baquero et al. 2009; Ramsayer et al. 2013). Interestingly, the evolution to tolerate elevated salinity conditions is a rather poorly studied topic in pathogenic bacteria, despite the migration of many species from freshwater to marine environments being a characteristic of coastal ecosystems. This is especially true when considering pathogens released to the natural environment from human-impacted areas, such as wastewater treatment plants. The latter contain bacteria such as our study species *S. marcescens* that is well known to be an opportunistic pathogen. Recently, *S. marcescens* strains originating from sewage were found to be primary cause of white pox disease in corals (Patterson et al. 2002). The consequences of these exotic pathogens for native species, aquaculture, fisheries, and human health are potentially enormous. Evolution in pathogen virulence and transmission is a widely studied field, but a pathogen's ability to withstand potentially adverse environmental conditions between the hosts might also be an important aspect of epidemiological dynamics. As salt tolerance evolved rapidly to a very high salt tolerance, *S. marcescens* cells that move to marine environments from freshwater ones could easily adapt to the new saline conditions, especially as the change in the salinity in natural conditions would be smaller than that tested here. Therefore, instead of sporadic epidemics of *S. marcescens*, one could see chronic epidemics in marine environments.

## Supporting Information

**Figure S1.** Weekly population biomasses (optical density) and salinities during the selection experiment in fluctuating environment treatments.

**Table S1.** Pairwise comparisons.
