# Supplementary material for: Rapid evolutionary adaptation to elevated salt concentrations in pathogenic freshwater bacteria Serratia marcescens
Source: Ecol Evol. 2014 Sep 23;4(20):3901–8. doi: 10.1002/ece3.1253 (PMC4242574; doi:10.1002/ece3.1253)
Supplement: Supplementary file 1 — Figure S1. Weekly population biomasses (optical density) and salinities during the selection experiment in fluctuating environment treatments. [file ece30004-3901-SD1.pdf]

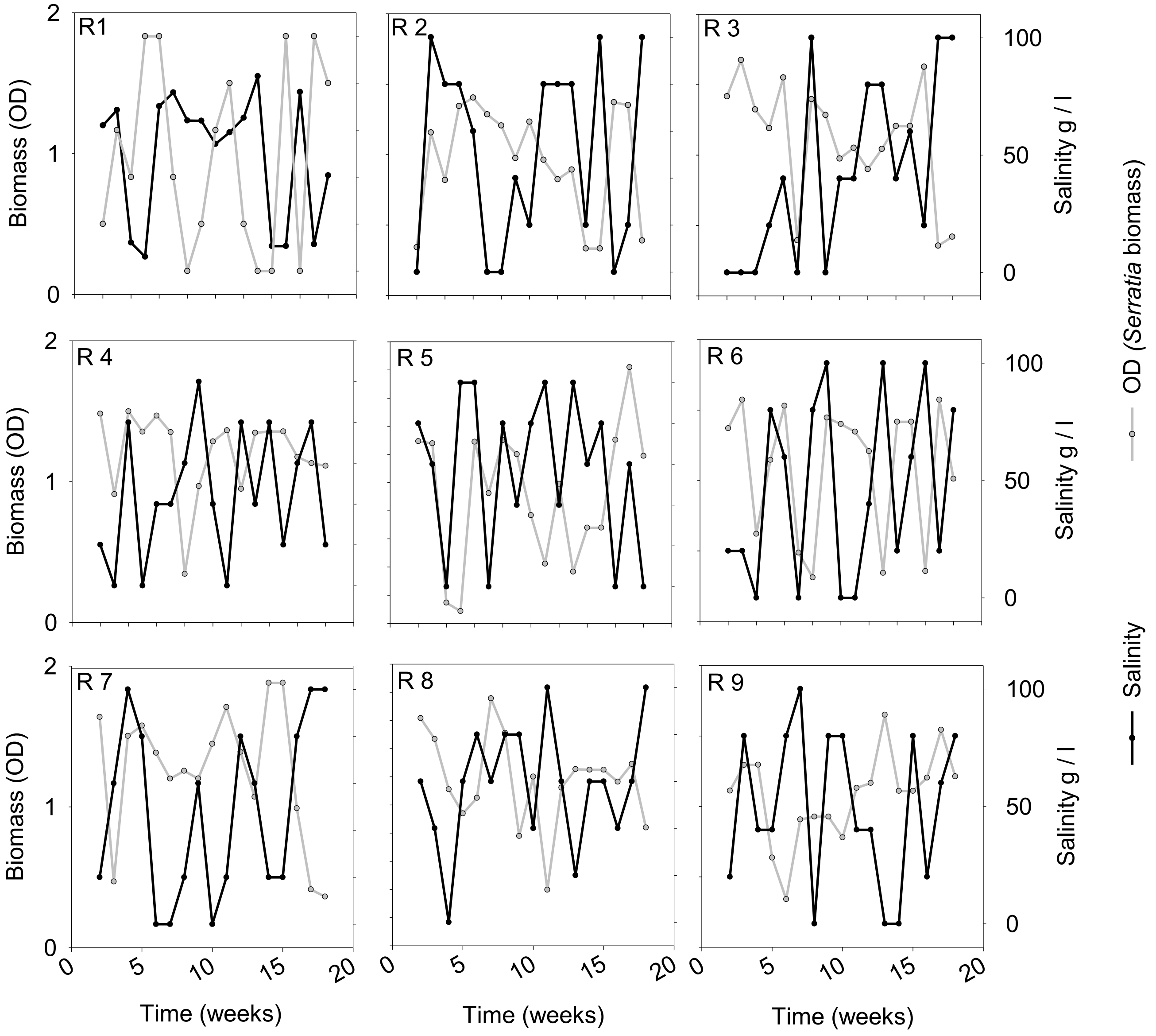

Supplement figure 1. Weekly population biomasses (optical density) and salinities during the selection experiment in fluctuating environment treatments. Each of the nine replicate populations had a different, randomly generated fluctuation type but with matching statistical properties. This design was adopted since week long exposures to certain environment could be visible in evolutionary outcome and if all replicate populations follow the same rhythm the changes in bacteria caused by past week's selection, could have been wrongly attributed to the evolutionary differences due to fluctuating regimes *per se*.
