# Supplementary material for: Rapid evolutionary adaptation to elevated salt concentrations in pathogenic freshwater bacteria Serratia marcescens
Source: Ecol Evol. 2014 Sep 23;4(20):3901–8. doi: 10.1002/ece3.1253 (PMC4242574; doi:10.1002/ece3.1253)
Supplement: Supplementary file 3 — Table S1. Pairwise comparisons. [file ece30004-3901-SD2.pdf]

Supplement table 1. Pairwise comparisons

Measurement Concentration	Evolutionary history (I)	(J)	M. Diff.	S.E	df	Sig.c	95% Con. Interval for Diff. Lower Bound	Upper Bound
0 g / l	ANC	0 g/l EVO	0.141	0.045	431.79	**0.017**	0.015	0.267
		80 g/l EVO	0.069	0.062	342.34	1	-0.106	0.244
		100 g/l EVO	-0.044	0.085	289.62	1	-0.286	0.197
		Fluctuating	0.132	0.045	435.77	**0.032**	0.006	0.258
	0 g/l EVO	ANC	-0.141	0.045	431.79	**0.017**	-0.267	-0.015
		80 g/l EVO	-0.072	0.058	280.08	1	-0.236	0.092
		100 g/l EVO	-0.185	0.083	257.2	0.255	-0.419	0.048
		Fluctuating	-0.009	0.039	419.46	1	-0.119	0.101
	80 g/l EVO	ANC	-0.069	0.062	342.34	1	-0.244	0.106
		0 g/l EVO	0.072	0.058	280.08	1	-0.092	0.236
		100 g/l EVO	-0.113	0.093	250.62	1	-0.377	0.15
		Fluctuating	0.063	0.058	315.27	1	-0.101	0.227
	100 g/l EVO	ANC	0.044	0.085	289.62	1	-0.197	0.286
		0 g/l EVO	0.185	0.083	257.2	0.255	-0.048	0.419
		80 g/l EVO	0.113	0.093	250.62	1	-0.15	0.377
		Fluctuating	0.176	0.083	273.45	0.334	-0.057	0.41
	Fluctuating	ANC	-0.1321	0.045	435.77	**0.032**	-0.258	-0.006
		0 g/l EVO	0.009	0.039	419.46	1	-0.101	0.119
		80 g/l EVO	-0.063	0.058	315.27	1	-0.227	0.101
		100 g/l EVO	-0.176	0.083	273.45	0.334	-0.41	0.057
70 g / l	ANC	0 g/l EVO	0.168	0.041	429.21	**0.001**	0.052	0.284
		80 g/l EVO	0.029	0.06	330.08	1	-0.14	0.199
		100 g/l EVO	-0.093	0.084	281.85	1	-0.331	0.144
		Fluctuating	0.119	0.042	435.9	**0.047**	0.001	0.236
	0 g/l EVO	ANC	-0.168	0.041	429.21	**0.001**	-0.284	-0.052
		80 g/l EVO	-0.139	0.058	279.04	0.167	-0.302	0.024
		100 g/l EVO	-0.261	0.082	256.55	**0.017**	-0.494	-0.029
		Fluctuating	-0.05	0.038	421.15	1	-0.158	0.059
	80 g/l EVO	ANC	-0.029	0.06	330.08	1	-0.199	0.14
		0 g/l EVO	0.139	0.058	279.04	0.167	-0.024	0.302
		100 g/l EVO	-0.123	0.093	250.62	1	-0.386	0.141
		Fluctuating	0.089	0.058	315.27	1	-0.075	0.253
	100 g/l EVO	ANC	0.093	0.084	281.85	1	-0.144	0.331
		0 g/l EVO	0.261	0.082	256.55	**0.017**	0.029	0.494
		80 g/l EVO	0.123	0.093	250.62	1	-0.141	0.386
		Fluctuating	0.212	0.083	273.45	0.108	-0.022	0.446
	Fluctuating	ANC	-0.119	0.042	435.9	**0.047**	-0.236	-0.001
		0 g/l EVO	0.05	0.038	421.15	1	-0.059	0.158
		80 g/l EVO	-0.089	0.058	315.27	1	-0.253	0.075
		100 g/l EVO	-0.212	0.083	273.45	0.108	-0.446	0.022
80 g / l	ANC	0 g/l EVO	0.151	0.042	426.24	**0.004**	0.032	0.271

		80 g/l EVO	-0.011	0.06	330.08	1	-0.18	0.159
		100 g/l EVO	-0.112	0.084	281.85	1	-0.349	0.125
		Fluctuating	0.126	0.042	435.9	**0.028**	0.008	0.243
	0 g/l EVO	ANC	-0.151	0.042	426.24	**0.004**	-0.271	-0.032
		80 g/l EVO	-0.162	0.059	281.22	0.061	-0.328	0.004
		100 g/l EVO	-0.263	0.083	257.93	**0.017**	-0.498	-0.029
		Fluctuating	-0.026	0.04	417.47	1	-0.138	0.086
	80 g/l EVO	ANC	0.011	0.06	330.08	1	-0.159	0.18
		0 g/l EVO	0.162	0.059	281.22	0.061	-0.004	0.328
		100 g/l EVO	-0.101	0.093	250.62	1	-0.365	0.162
		Fluctuating	0.136	0.058	315.27	0.196	-0.028	0.3
	100 g/l EVO	ANC	0.112	0.084	281.85	1	-0.125	0.349
		0 g/l EVO	0.263	0.083	257.93	**0.017**	0.029	0.498
		80 g/l EVO	0.101	0.093	250.62	1	-0.162	0.365
		Fluctuating	0.237	0.083	273.45	**0.043**	0.004	0.471
	Fluctuating	ANC	-0.126	0.042	435.9	**0.028**	-0.243	-0.008
		0 g/l EVO	0.026	0.04	417.47	1	-0.086	0.138
		80 g/l EVO	-0.136	0.058	315.27	0.196	-0.3	0.028
		100 g/l EVO	-0.237	0.083	273.45	**0.043**	-0.471	-0.004
90 g / l	ANC	0 g/l EVO	0.158	0.041	429.21	**0.001**	0.042	0.274
		80 g/l EVO	-0.168	0.06	330.08	0.053	-0.338	0.001
		100 g/l EVO	-0.117	0.084	281.85	1	-0.354	0.121
		Fluctuating	0.042	0.042	435.9	1	-0.075	0.16
	0 g/l EVO	ANC	-0.158	0.041	429.21	**0.001**	-0.274	-0.042
		80 g/l EVO	-0.327	0.058	279.04	**<0.001**	-0.49	-0.164
		100 g/l EVO	-0.275	0.082	256.55	**0.009**	-0.508	-0.042
		Fluctuating	-0.116	0.038	421	**0.026**	-0.224	-0.008
	80 g/l EVO	ANC	0.168	0.06	330.08	0.053	-0.001	0.338
		0 g/l EVO	0.327	0.058	279.04	**<0.001**	0.164	0.49
		100 g/l EVO	0.052	0.093	250.62	1	-0.212	0.315
		Fluctuating	0.211	0.058	314.99	**0.003**	0.047	0.375
	100 g/l EVO	ANC	0.117	0.084	281.85	1	-0.121	0.354
		0 g/l EVO	0.275	0.082	256.55	**0.009**	0.042	0.508
		80 g/l EVO	-0.052	0.093	250.62	1	-0.315	0.212
		Fluctuating	0.159	0.083	273.3	0.547	-0.074	0.393
	Fluctuating	ANC	-0.042	0.042	435.9	1	-0.16	0.075
		0 g/l EVO	0.116	0.038	421	**0.026**	0.008	0.224
		80 g/l EVO	-0.211	0.058	314.99	0.003	-0.375	-0.047
		100 g/l EVO	-0.159	0.083	273.3	0.547	-0.393	0.074
100 g / l	ANC	0 g/l EVO	0.117	0.044	432.83	0.08	-0.007	0.241
		80 g/l EVO	-0.016	0.063	342.44	1	-0.193	0.162
		100 g/l EVO	-0.086	0.085	289.62	1	-0.327	0.156
		Fluctuating	0.067	0.044	435.76	1	-0.059	0.192
	0 g/l EVO	ANC	-0.117	0.044	432.83	0.08	-0.241	0.007
		80 g/l EVO	-0.133	0.059	281.29	0.241	-0.299	0.033
		100 g/l EVO	-0.203	0.082	256.55	0.142	-0.436	0.03

	Fluctuating	-0.051	0.038	420.9	1	-0.158	0.057
80 g/l EVO	ANC	0.016	0.063	342.44	1	-0.162	0.193
	0 g/l EVO	0.133	0.059	281.29	0.241	-0.033	0.299
	100 g/l EVO	-0.07	0.094	251.8	1	-0.335	0.195
	Fluctuating	0.082	0.059	315.85	1	-0.084	0.249
100 g/l EVO	ANC	0.086	0.085	289.62	1	-0.156	0.327
	0 g/l EVO	0.203	0.082	256.55	0.142	-0.03	0.436
	80 g/l EVO	0.07	0.094	251.8	1	-0.195	0.335
	Fluctuating	0.152	0.082	273.18	0.657	-0.081	0.386
Fluctuating	ANC	-0.067	0.044	435.76	1	-0.192	0.059
	0 g/l EVO	0.051	0.038	420.9	1	-0.057	0.158
	80 g/l EVO	-0.082	0.059	315.85	1	-0.249	0.084
	100 g/l EVO	-0.152	0.082	273.18	0.657	-0.386	0.081

Based on estimated marginal means

* The mean difference is significant at the .05 level.

a Dependent Variable: K.

c Adjustment for multiple comparisons: Bonferroni.
